# K acquisition from vermiculite by sweet potato also improves K nutrition in neighboring plants

**DOI:** 10.1038/s41598-026-56682-4

**Published:** 2026-06-10

**Authors:** Fan Mo, Yan Yi, Katsuya Yano

**Affiliations:** 1 Laboratory of Crop Science, Graduate School of Bioagricultural Sciences, Nagoya University, Furo-cho, Chikusa-ku, Nagoya, Aichi 464-0814 Japan; 2 Panxi Crops Research and Utilization Key Laboratory of Sichuan Province, School of Agricultural Science, Xichang University, Xichang, Sichuan 615013 China; 3 International Research Fellow of Japan Society for the Promotion of Science, Tokyo, 102-0083, Japan

**Keywords:** *Ipomoea batatas*, *Glycine max*, Vermiculite, X-ray fluorescence mapping (XRF), Rhizosphere, Ecology, Ecology, Environmental sciences, Plant sciences

## Abstract

Intensive agriculture requires substantial external potassium (K) application to meet crop K requirements. Recent research has revealed that sweet potato (*Ipomoea batatas*), a crop with high K requirements, could acquire sufficient K from a mixture of Andosol and vermiculite without the need for additional fertilization. However, the specific K component in growth medium being utilized is still unclear. We evaluated K acquisition by sweet potato grown in either Andosol or vermiculite, comparing it with soybean, barley and water spinach (*Ipomoea aquatica*). Additionally, soybeans were grown in vermiculite together with sweet potato to assess the rhizosphere effect of sweet potato. Changes in K component in growth medium after plant growth were examined to identify the specific fraction responsible for these changes. Only sweet potato and water spinach, both from the genus *Ipomoea*, exhibited normal growth in vermiculite-only medium without K application by promoting the release of K from slowly available (fixed interlayer K) pools, which is unavailable to other crop species. K mapping image analysis confirmed that sweet potato roots accumulated more K than other species, even during 7 days of growth. In the mixed-culture system, the presence of sweet potato enhanced K availability in vermiculite to soybean (*Glycine max*), enhancing K and biomass accumulation of soybean. Our results reveal that sweet potato and water spinach exhibit efficient K acquisition from vermiculite, especially from slowly available K pool. Besides, sweet potato enhances K availability to neighboring plants through rhizosphere acidification.

**Supplementary Information:**

The online version contains supplementary material available at 10.1038/s41598-026-56682-4.

## Introduction

Next to nitrogen (N), potassium (K) has the second highest concentration in plant tissues, typically ranging from 20 to 50 mg K g^−1^ dry matter^1^. K plays a crucial role in photosynthesis^2^, stomatal regulation^3^, phloem transport^4^, and salinity tolerance^5^. In intensive agriculture, large amounts of K fertilizers are applied to meet plant K requirements^6^. However, their use can be inefficient, and under certain conditions (e.g., absence of cover crops), 4–32% of applied K may be lost through rainfall and irrigation^7^. Besides, the high cost and transportation expenses of conventional K fertilizer hinder agricultural production in developing countries^8,9^. Thus, exploring alternative K resources has become an urgent priority in light of the unstable supply and potential depletion of potash reserves.

In this context, sweet potato (*Ipomoea batatas*) presents an interesting planCharacteristics and K ct, as it may utilize K sources in the soil that are unavailable to other plant species. Although tuberous crops, including sweet potato, are generally considered to have a higher K requirement than other crops^10^, several studies have demonstrated that sweet potato can achieve high yields without external K inputs^10,11^. Conventionally, the strong K acquisition capacity of sweet potato in low-K soils is attributed to the increased activity of High-Affinity Potassium (HAK) transporters within its roots^12,13^. However, the potential rhizosphere effect of sweet potato might be overlooked. Ning et al.^14^ have reported that water spinach (*Ipomoea aquatica*) enhanced soil nutrient conditions through rhizosphere acidification by root proton efflux. As a close relative, the possibility that sweet potato (*Ipomoea batatas*) employs a similar rhizosphere mechanism to enhance soil K availability warrants further investigation.

More than 99.9% of total soil K is present in three distinct pools, each differing in their accessibility to plant roots^15^. Exchangeable K, typically adsorbed on the soil particles and extracted with ammonium acetate, is considered a readily available K source for most plant species, while it only takes up around 2% of soil K component^16^. In contrast, fixed K (often referred to as non-exchangeable K) refers to potassium fixed within interlayers of clay minerals such as vermiculite and smectite, is thought to have limited bioavailability, depending on soil mineralogy and plant species^17,18^. Structural K, incorporated into the crystal structure of primary minerals, is hardly available, with its release typically occurring over extended periods, often requiring years. Recently, Ueda and Yano^19^ have found that K accumulation in sweet potato was unaffected by K-fertilizer application in a mixture of Andosol and vermiculite, with similar K accumulation observed even in the absence of K fertilization. However, because the media were combined, it was impossible to determine whether sweet potato was utilizing extra K source directly from the vermiculite. To deconstruct this observation and clarify the K source in the growth medium, the present study was designed to evaluate the K acquiring capacity of sweet potato.

In this study, we conducted pot experiments to assess K acquisition by sweet potato (*Ipomoea batatas*, cv. Beniazuma) grown in either Andosol or vermiculite, compared with soybean (*Glycine max*), barley (*Hordeum vulgare*), and water spinach. We focused on changes in chemical K fractions such as exchangeable, non-exchangeable, and slowly available K in each medium after plant growth to determine whether sweet potato can utilize a unique K source unavailable to other species. Additionally, we examined whether K acquisition by a plant with low K acquisition efficiency in a given medium, can be improved when co-cultured with sweet potato. If significant improvement is observed, the involvement of rhizosphere effect acting outside the roots of sweet potato, would be implied.

The objectives of the current study were to address the following questions:

1. Do plants utilize vermiculite as a K source?
2. Is such K-utilizing capability unique to sweet potato among the species examined?
3. Does co-cultivation with sweet potato improve K accumulation in soybean with poor K acquisition?

## Materials and methods

### Experimental design

#### Growth response of four plant species in mixed soil medium and vermiculite-only medium in 2023

In 2023, growth response experiments were conducted under an open-sided rain-shelter greenhouse that protected pots from precipitation while maintaining ambient temperature, humidity, and light conditions at the Higashiyama Campus of Nagoya University (35.16°N, 136.97°E), Nagoya, Japan. The experiment included five treatments: four mixed soil treatments and one pure vermiculite treatment. For the mixed soil treatments, each pot (24 cm diameter, 23 cm depth) was filled with 6 L of a 1:1 (v/v) autoclaved mixture of Andosol (29% sand, 55% silt, and 16% clay; pH 6.1; total C 89 g kg^−1^, total N 7.2 g kg^−1^) and commercial vermiculite (Nitto vermiculite Co.) (Table S1). For the vermiculite-only treatment, pot in same size (24 cm diameter, 23 cm depth) contained 6 L of vermiculite. The mixed soil stay consistent with previous work^19^. In contrast, the vermiculite-only medium was selected specifically as a challenging growth medium to test the plant K acquisition capacity, as it is commonly characterized as a soil water-retention amendment and higher ratio of non-exchangeable K (Table 1). Each pot was irrigated from the bottom using a 30 cm diameter, 4 cm deep individual sub-irrigation tray, with the water level maintained at 1 cm throughout the 60 days experimental period with distilled water.

**Table 1:** Initial characteristics of growth medium before transplanting.

Growth medium	Soil pH	Density (kg L^−1^)	Total K content (g K kg^−1^)	Exchangeable K concentration (cmol kg^−1^)	Non-exchangeable K concentration (g K kg^−1^)	Exchangeable K content (g pot^−1^)	Non-exchangeable K content (g pot^−1^)
Andosol	6.3	0.95	n.a.	0.31	0.54	n.a.	n.a.
Mixed soil (Andosol: vermiculite = 1: 1 v/v)	6.1	0.58	31.13	0.51	1.46	0.70	5.08
Vermiculite-only	7.0	0.22	34.32	1.18	5.21	0.61	6.88

In all treatments, 1.1 g of urea (46% N) and 30 g of superphosphate (7.5% P) were applied at uniform rates and mixed with the growth medium. Four levels of K fertilization (0, 0.5, 1, 2 g pot^−1^) using potassium chloride (50% K) were applied at varying rates and uniformly mixed with the growth medium. The maximum rate of 2 g pot^−1^ was intentionally selected to exceed the highest rate (1.6 g pot^−1^) used in previous studies^19^, ensuring a sufficiently wide range to determine if previous non-responses were due to insufficient K application. Notably, no visible symptoms of chloride toxicity were observed across any treatments. No K fertilization was applied to vermiculite-only treatment. Sweet potato shoots (sterilized, approximately 40 cm in length) and 10 day-old sterilized seedlings (immersed in 70% ethanol for 30 s, followed by 1% sodium hypochlorite (NaClO) for 5 min, and rinsed three times with sterile distilled water) of water spinach, soybean, and barley were transplanted on May 15, 2023. Seeds and seedlings were surface-sterilized by immersion in 70% (v/v) ethanol for 1 min, followed by 1% (w/v) sodium hypochlorite containing 0.05% Tween 20 for 10 min, and then rinsed five times with sterile distilled water. To maintain spatial and total biomass equivalency, sweet potato and water spinach were planted at one seedling per pot, whereas soybean and barley were planted at three and five seedlings per pot, respectively. To investigate the K accumulation of plants during the growth period, the initial K content was determined by destructively measure the K content of different plant seedlings (*n* = 5) at the time of transplanting. The average initial K content per plant was 0.37 ± 0.04 g for sweet potato, 0.0007 ± 0.0002 g for water spinach, 0.03 ± 0.01 g for soybean, and 0.0003 ± 0.0001 g for barley. All plants were harvested 60 days after transplanting to minimize confounding variables brought by plant senescence.

#### X-ray fluorescence mapping analysis

X-ray fluorescence mapping tests were conducted using sweet potato, soybean, barley, and water spinach. Two fresh sweet potato shoots were grown in the field at Higashiyama Campus in Nagoya University. The shoots were then cut to a length of approximately 7 cm and inserted into a pot filled with vermiculite, where they were kept in a growth chamber for 7 days to promote root formation. Soybean, barley, and water spinach seeds were sown in seedling trays filled with vermiculite (25 mm length, 25 mm width, 45 mm depth), with one seed placed per hole. The vermiculite surface was kept moist for 10 days until the seedlings emerged. The plant roots were carefully washed and transplanted into two foam plastic boxes (10 cm in length, 10 cm width, 3.5 cm depth), each filled with vermiculite. The boxes were divided into four separate compartments using 3 mm thick foam boards, which were stuck together with synthetic resins (EVA resin and petroleum resin) to prevent roots penetration through the gaps. The boxes were then placed in a growth chamber for 7 days, with the vermiculite kept moist throughout.

After 7 days of growth, all the above-ground parts were cut off. The bottom foam boards were removed from the upside-down foam plastic box to exposure the rooted vermiculite. An X-ray analytical microscope (XGT-5000, HORIBA, Japan) was used to create an X-ray fluorescence map, visualizing the distribution of K and other nutrient elements (Fe, S, Na, etc.) on the vermiculite medium and root surfaces. The X-ray settings were as follows: tube voltage 50kV, current 100mA, beam spot size 10 μm, pulse processing time P1, transmitted X-ray intensity X7, and a cumulative total of 60 X-ray image pickups. Mapping area was 100mm × 100mm at a pixel resolution of 10 μm. Element maps were generated using the manufacturer’s software (HORIBA XGT analysis software, HORIBA, Japan) running on Windows XP, which displays signal intensity as counts per pixel (cps) on a calibrated color scale.

#### Mixed-culture experiment in a vermiculite-only medium in 2024

In 2024, a mixed-culture experiment was conducted at the Higashiyama Campus of Nagoya University, Nagoya, Japan. Two mixed-culture combinations (sweet potato & soybean, soybean & soybean) were set in this experiment. For all treatments, pots (24 cm diameter, 23 cm depth, water permeable, recycled polypropylene) were filled with 6 L of vermiculite. A total of 1.1 g of urea (46% N) and 30 g of superphosphate (7.5%P) were applied in each pot as in the growth response experiment in 2023. No external K fertilizer was applied. On June 1, 10 days-old soybean seedlings were transplanted into pots with a 15 cm spacing between plants. All plants were harvested 60 days after transplanting. To maintain spatial and total biomass equivalency, sweet potato were planted at one seedling per pot, whereas soybean were planted at three per pot.

Four replications were prepared for each treatment. To eliminate the effect of growth medium moisture, the plants were maintained under well-watered conditions throughout the growth period. Each pot was irrigated from the bottom using a 30 cm diameter, 4 cm deep individual sub-irrigation tray, with the water level maintained at 1 cm throughout the 60 days experimental period with distilled water. The water level of trays was manually checked on three occasions per day, at 8:00 a.m., 13:00 p.m., and 6:00 p.m., respectively. Irrigation was conducted when the water level is below 1 cm to maintain same water conditions for each treatment. At sampling, sweet potato and soybean shoots were sampled as a whole. Roots and tubers were carefully washed with tap water to remove growth medium. The samples were dried in an oven at 80°C until reaching a constant mass to determine dry weight and then ground for further analysis.

#### K quantification of plant materials

The ground samples from each organ were extracted by 15 mL of 1 mol L^−1^ HCl by shaking for 1 hour^19^. The solution was then left overnight and filtered through filter paper (No. 6, 90 mm diameter, ADVANTEC Inc., Japan). K concentration was determined by analyzing aliquots of the filtrate using an atomic absorption spectrophotometer (AA-6200, Shimadzu corp., Japan).

#### K quantification in growth medium

Approximately 20 g of homogenized growth medium (avoiding vertical variation) was collected for analysis before the experiment began and after 60 days of growth. The collected samples were dried in an oven at 80°C and then passed through a 2.00 mm sieve. Exchangeable K was extracted using 1 mol L^−1^ CH_3_COONH_4_^20^. A separate sample of growth medium was extracted with 1 mol L^−1^ boiling HNO_3_. Non-exchangeable K was calculated as the difference between K extracted by 1 mol L^−1^ CH_3_COONH_4_ and 1 mol L^−1^ CH_3_COONH_4_^21^. The extracted solutions were then filtered through filter paper (No. 6, 90 mm diameter, ADVANTEC Inc., Japan). Aliquots of the filtrate were then analyzed using an atomic absorption spectrophotometer to determine K concentration.

#### Determination of pH value in growth medium

pH value was measured from a suspension of 1:2.5 soil: distilled water^22^ with a pH meter (D-73, HORIBA Co., Japan).

### Calculation of changes in slowly available K in growth medium

In this research, non-exchangeable K is defined as the difference between K extracted by 1 mol L^−1^ NHO_3_ and that extracted by 1 mol L^−1^ CH3COONH_4_^21^. As the release of fixed K is highly dependent on the extractant chemistry and extraction duration^23^, the actual pool of interlayer K potentially available to plants in the growth medium might exceed what this specific chemical extraction captures. Under these circumstances, changes in slowly available K (total available K mobilized other than exchangeable K) in growth medium were calculated to describe utilized interlayer K by plant depletion through comparing the K balance before and after 60 days of growth.

1 $$\begin{array}{*{20}c} {{\mathrm{Changes}}\; {\mathrm{in}} \;{\mathrm{slowly}}\; {\mathrm{available}} K} \\ { = {\mathrm{Fertilizer}} K {\mathrm{input}} - {\mathrm{Plant}} K {\mathrm{removal}} - {\mathrm{Changes}}\; {\mathrm{in}}\; {\mathrm{exchangeable}} K} \\ \end{array}$$

where $Fertilizer K input$ represents the K added through fertilizer K application. *Plant K removal* was calculated by multiplying the total biomass of the plant by its K concentration after 60 days of growth, then subtracting the initial K content before transplanting. The experiment was conducted in controlled pot conditions over a 60 day period. Given the short duration and the contained environment with sub-irrigation trays that recaptured any leachate, potential nutrient losses due to leaching were considered negligible and were not accounted for in this study.

To be noticed, two limitations of our mass balance approach should be noted. First, only end-point measurements were collected. While intermediate time-point data, which would allow direct validation of mass balance closure during the 60 day growth period, were not obtained. Second, although the sub-irrigation tray design minimized lateral loss of K, small losses such as K retained in the tray cannot be fully excluded. Any unaccounted losses would be attributed to either plant K removal or to the slowly available K pool, potentially inflating the latter. However, the interspecific contrast between Ipomoea species and soybean/barley in vermiculite cannot be explained by such losses, which would affect all species equally. Due to vermiculite’s fixation capacity and the limits of chemical extraction, our K mass balance calculations should be viewed as estimates of K movement rather than exact, absolute measurements.

### Root morphology

After the mixed-culture experiment, the plant roots were carefully washed. Fresh roots were scanned at a resolution of 600 dpi using a scanner (GT-X980, Seiko Epson Co., Japan). The scanned images were processed using RhizoVision Explorer software^24^ to obtain root length and surface area. The software was configured with the following settings: image thresholding level set to 200, non-root objects filtered out with a maximum size of 1 pixel, and edge smoothing threshold set to level 2. The plant roots were then dried to a constant weight at 80 °C to determine root biomass. Root length or surface area production per unit dry mass were described in terms of specific root length (SRL,m g^−1^) or specific root area separately (SRA; m^2^ g^−1^)^25^. The root to shoot ratio was calculated as the ratio of belowground biomass to aboveground biomass^26^.

### Vermiculite K-release response to pH

To investigate the potential effect of proton concentration on the availability of exchangeable potassium (K) in vermiculite under rhizosphere conditions, a soil incubation experiment was conducted. For each treatment, 5 g of vermiculite was placed into glass vials and incubated with 50 mL of buffer solution composed of Na_2_HPO_4_ and Na_2_HPO_4_. Three pH levels were established (6.0, 6.5, and 7.0), reflecting the rhizosphere pH conditions observed in the mixed-culture experiment. The samples were incubated at room temperature for 24 hours, with three replicates per treatment. After incubation, the vermiculite was oven-dried for 12 hours, and exchangeable K was subsequently extracted and measured.

### Statistical analysis

Pot experiments were conducted using a randomized block design with four biological replicates per treatment, and data are presented as mean ± standard deviation (SD). Prior to the application of parametric tests, data distributions and residual patterns were inspected to confirm the suitability of analysis of variance (ANOVA) for each dataset. Where the experimental design involved a single factor, one-way ANOVA was applied. Where two factors were tested simultaneously, two-way ANOVA was used to assess both main effects and their interaction. When significant main effects or interactions were detected at *P* < 0.05, pairwise comparisons among treatment means were performed using Tukey’s honestly significant difference (HSD) post hoc test, which incorporates correction for multiple comparisons and controls the family-wise error rate. For the mixed-culture experiment (2024), in which only two treatment groups were compared, independent samples t-tests were applied. For the buffer incubation experiment, differences in exchangeable K release among the three pH levels were tested using one-way ANOVA followed by Tukey’s HSD post hoc test. All statistical analyses were performed using the SciPy library^27^ in Python. Coefficient of variation (CV) values for each treatment cell of Table 2, providing an additional measure of relative variability, are reported in Supplementary Table S3. All figures were generated with OriginPro (Version 2023, OriginLab Corporation, Northampton, MA, USA).

**Table 2:** Characteristics and K component of growth medium at different fertilizer K inputs after 60 days of growth in greenhouse with mixed soil medium in growth response experiment in 2023.

Species	Treatment (g K pot^−1^)	pH value	Exchangeable K concentration (cmol kg^−1^)	Exchangeable K content (g pot^−1^)	Non-exchangeable K content (g pot^−1^)	Changes in slowly available K pool^†^ (g pot^−1^)
Sweet potato	0.00	5.48±0.06	0.39±0.04	0.53±0.05	4.42±1.03	−1.63±0.34
	0.25	5.58±0.04	0.47±0.08	0.64±0.11	4.24±0.64	−1.29±0.20
	0.50	5.64±0.07	0.57±0.08	0.77±0.11	5.09±1.68	−1.29±0.19
	1.00	5.63±0.06	0.51±0.07	0.69±0.10	5.40±2.06	−0.89±0.26
One-way ANOVA		*P* = 0.009	*P* = 0.026	*P* = 0.026	*P* = 0.653	*P* = 0.012
Water spinach	0.00	5.56±0.09	0.51±0.08	0.69±0.11	4.61±0.82	−1.26±0.32
	0.25	5.53±0.08	0.52±0.02	0.71±0.03	4.81±1.74	−1.67±0.35
	0.50	5.50±0.03	0.53±0.05	0.72±0.07	5.32±1.94	−1.55±0.29
	1.00	5.51±0.06	0.64±0.04	0.87±0.06	4.77±0.47	−1.85±0.07
One-way ANOVA		*P* = 0.662	*P* = 0.015	*P* = 0.015	*P* = 0.897	*P* = 0.066
Barley	0.00	5.66±0.03	0.66±0.06	0.90±0.08	5.90±0.46	−0.71±0.07
	0.25	5.63±0.10	0.74±0.02	0.99±0.03	5.21±0.43	−0.80±0.50
	0.50	5.68±0.08	0.65±0.08	0.89±0.11	5.41±0.60	−0.49±0.49
	1.00	5.61±0.09	1.04±0.34	1.42±0.46	5.46±0.50	−0.61±0.51
One-way ANOVA		*P* = 0.659	*P* = 0.027	*P* = 0.027	*P* = 0.298	*P* = 0.766
Soybean	0.00	5.98±0.13	0.45±0.14	0.61±0.19	4.84±1.39	−1.43±0.20
	0.25	6.04±0.12	0.52±0.12	0.71±0.16	4.75±0.98	−1.38±0.13
	0.50	5.83±0.55	0.55±0.07	0.75±0.09	5.18±1.12	−1.17±0.09
	1.00	6.05±0.15	0.53±0.15	0.72±0.21	4.57±0.62	−0.77±0.30
One-way ANOVA		*P* = 0.651	*P* = 0.680	*P* = 0.680	*P* = 0.875	*P* = 0.002
Two-way ANOVA	K input	*P* = 0.309	*P* < 0.001	*P* < 0.001	*P* = 0.408	*P* = 0.077
	Species	*P* < 0.001	*P* < 0.001	*P* < 0.001	*P* = 0.935	*P* < 0.001
	K input × Species	*P* = 0.304	*P* = 0.033	*P* = 0.033	*P* = 0.768	*P* = 0.008

^†^Changes in slowly available K pool were calculated as described in Equ. (1).

*P*-values from a one-way ANOVA testing the effect of K fertilizer rate within that specific species. *P*-values from a two-way ANOVA testing the main effects of K input, Species, and their interaction across all treatments. For all analyses, effects were considered statistically significant at *P* < 0.05, and corresponding *p*-values are highlighted in bold.

## Results

### Growth responses of different plant species to fertilizer K inputs in 2023

**Fig. 1:**
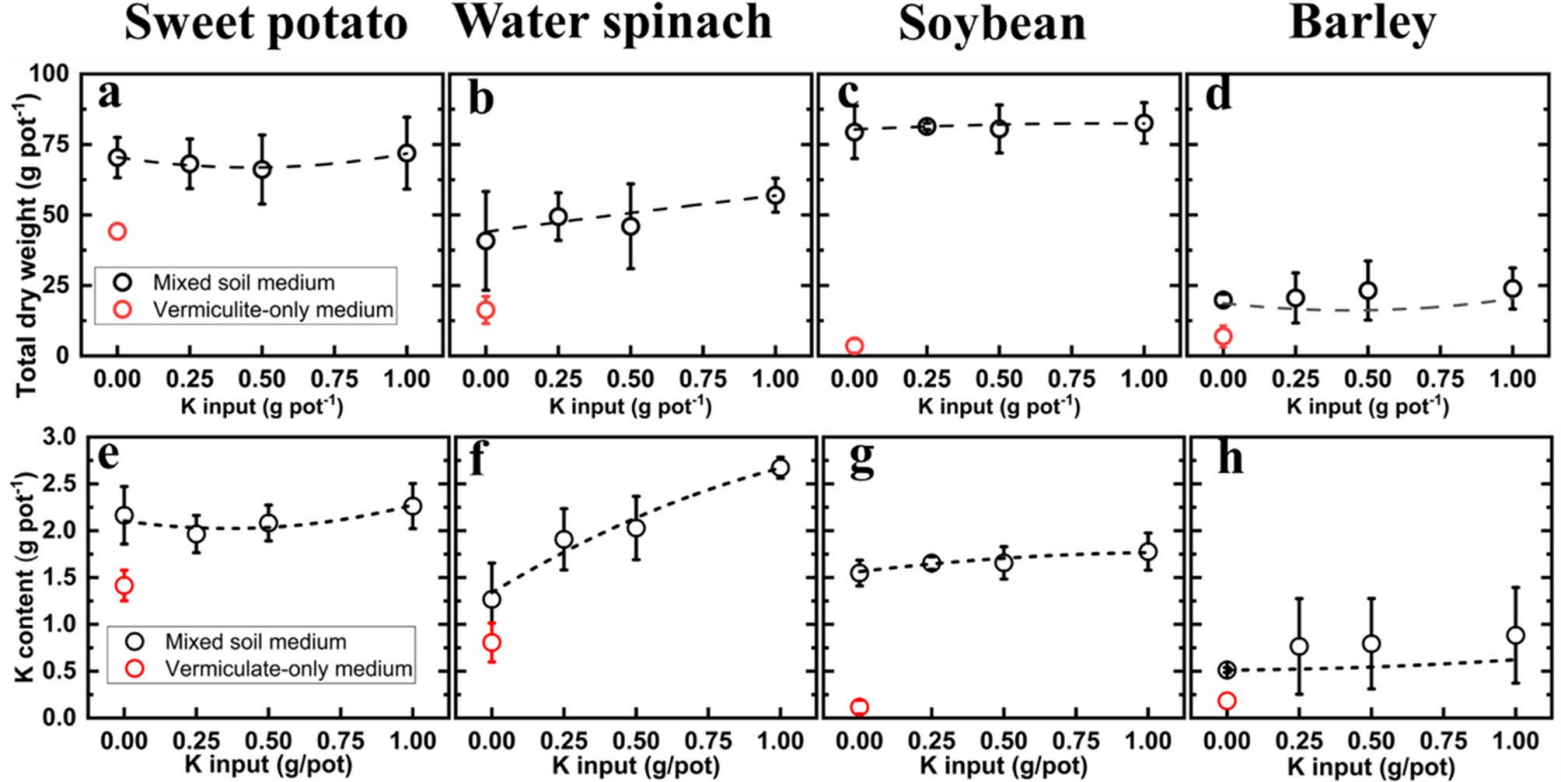
Biomass (**a**–**d**) and K content (**e**–**h**) in sweet potato, soybean, water spinach, and barley grown in greenhouse with the mixed soil medium and vermiculite-only condition in growth response experiment in 2023 after 60 days of growth. (**a**, **e**) Sweet potato, (**b**, **f**) water spinach, (**c**, **g**) soybean, and (**d**, **h**) barley. Black circles represent the mixed soil medium, and red circles represent the vermiculite-only condition. Error bars represent standard deviation (*n*=4). Dashed lines are added to guide the eye and do not represent a fitted model.

To elucidate the responses of different plant species to additional K inputs, plant biomass and K content were measured after 60 days of growth in mixed soil medium (Fig. 1a–h). K input did not increase plant biomass across species (Fig. 1a–d), but interspecific variability in both plant biomass and K content was observed (Fig. 1a–h). K content of sweet potato, barley, and soybean showed no response to added K, with maximum growth occurring at 0 g K pot^−1^ fertilizer input (Fig. 1e, g, h).

In the vermiculite-only medium, sweet potato and water spinach maintained normal growth, accumulating 1.05 g K pot^−1^ and 0.80 g K pot^−1^, respectively, after 60 days (Fig. 1e, f). In contrast, barley and soybean accumulated less than 0.2 g K pot^−1^ (Fig. 1g, h) and failed to sustain normal growth (Fig. S1).

#### Characteristics and K component of growth medium after plant growth in growth response experiment in 2023

Table 2 shows that different plant species exhibited distinct patterns of exchangeable K utilization in the mixed soil medium. Sweet potato and soybean demonstrated strong access to exchangeable K in the absence of K fertilization. Exchangeable K dropped to 0.39 and 0.45 cmol pot^−1^, leaving 0.53 and 0.61 g K pot^−1^ exchangeable K content in the growth medium, respectively. Moreover, additional K application significantly increased the exchangeable K content in the growth medium (*P* < 0.001). In contrast, the utilization of non-exchangeable was not significantly influenced by plant species, K fertilization, or their interaction effects (Table 2).

Table 2 shows interspecific differences in pH value in the mixed soil medium after 60 days of plant cultivation. In treatments without K fertilizer, the cultivation of sweet potato and water spinach resulted in a lower pH value (5.48 and 5.56, respectively) in growth medium compared to barley and soybean. For sweet potato, increasing the K application rate significantly raised the final pH value (*P* = 0.009). In contrast, K inputs had no significant effect on pH value of growth medium when grown with water spinach, barley, or soybean.

In the vermiculite-only medium, no significant differences were observed in the exchangeable K content across the different plant species after 60 days of growth (*P* = 0.115) (Table 3). Interspecific differences were found in the changes in the slowly available K pool, with sweet potato and water spinach exhibiting higher levels of K acquisition (Table 3) and root K concentration (Table S2).

**Table 3:** Soil characteristics and K components across different plant species after 60 days of growth in vermiculite-only medium without K fertilization.

Species	Exchangeable K concentration (cmol kg^−1^)	Exchangeable K content (g pot^−1^)	Non-exchangeable K content (g pot^−1^)	Changes in slowly available K pool^†^ (g pot^−1^)
Sweet potato	0.89±0.10	0.46±0.05	6.47±0.61	−0.90±0.18
Water spinach	0.95±0.23	0.49±0.12	6.59±0.53	−0.69±0.31
Barley	1.03±0.02	0.53±0.01	8.18±0.79	−0.10±0.10
Soybean	0.89±0.58	0.46±0.30	6.65±0.75	0.07±0.12
ANOVA	*P* = 0.115	*P* = 0.115	*P* = 0.048	*P* < 0.001

^†^Changes in slowly available K pool were calculated as described in Equ. (1).

For all analyses, effects were considered statistically significant at *P* < 0.05, and corresponding *p*-values are highlighted in bold.

**Fig. 2:**
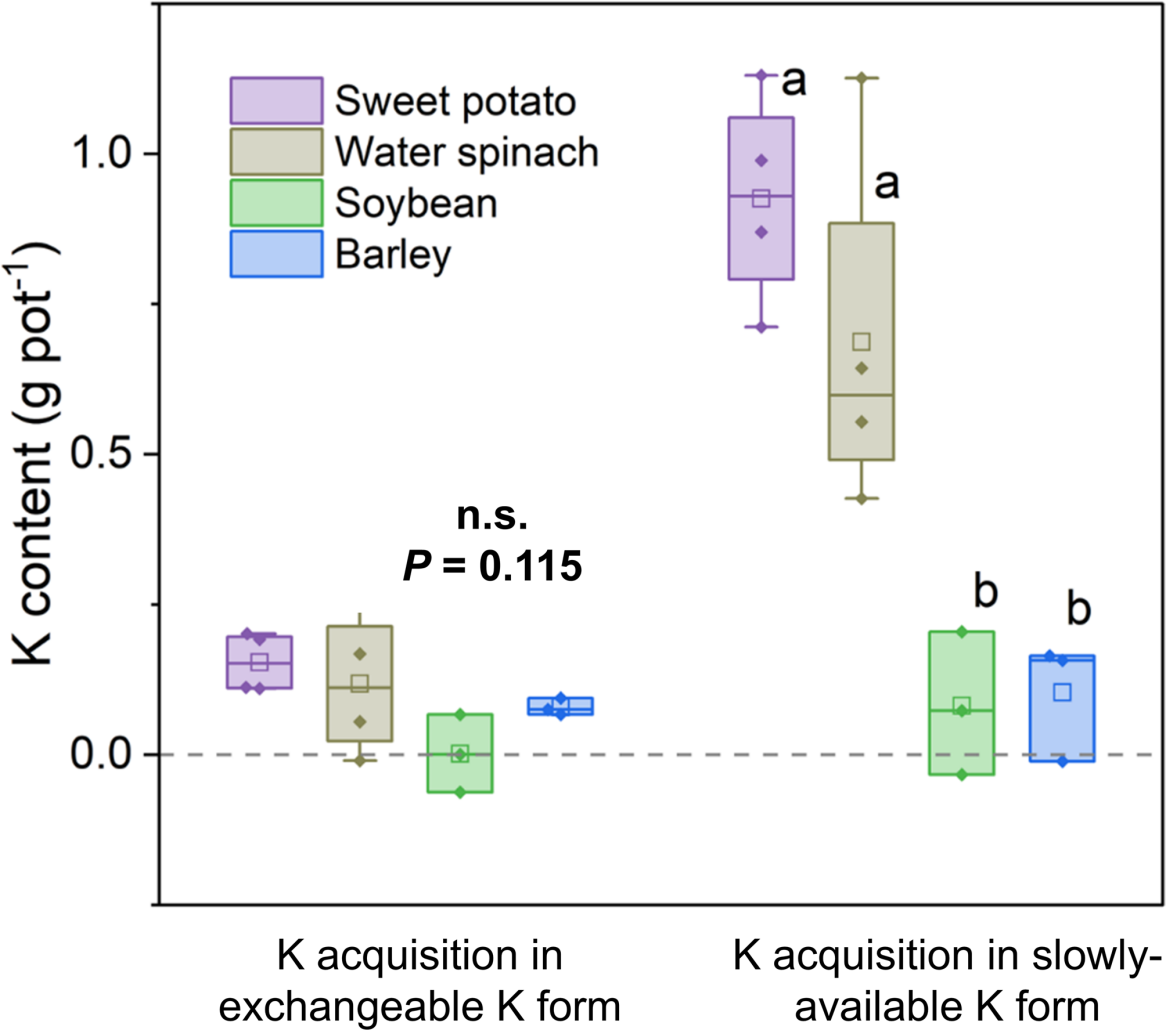
Plant K acquisition in sweet potato, barley, water spinach, and soybean from different K components of vermiculite in growth response experiment in 2023 with vermiculite-only medium. Data are shown as box plots, where the box represents the interquartile range (IQR), the horizontal line is the median, and the square is the mean. K acquisition from the exchangeable K pool was calculated as the net change in exchangeable K content in growth medium over the 60 day growth period. K acquisition from the slowly available pool was calculated as the difference between the total K accumulated by the plant and the K acquired from the exchangeable pool. Statistical comparisons among the four plant species were performed independently for each K acquisition form using a one-way ANOVA. For the exchangeable K form, the overall *P*-value is shown, with 'n.s.' indicating no significant difference. For the slowly-available K form, different letters (**a**, **b**) denote statistically significant differences among the plant species according to a Tukey’s honestly significant difference (HSD) post-hoc test (*P* < 0.05).

Exchangeable K acquisition capacity among the different species was not significantly different (Fig. 2). The significant K uptake by sweet potato and water spinach was sustained by a large release of K from the slowly available pool to replenish the exchangeable K pool. A mass balance calculation shows that this replenishment ultimately accounted for over 85% of the total K accumulated by these plants (Fig. 2).

#### X-ray fluorescence mapping

**Fig. 3:**
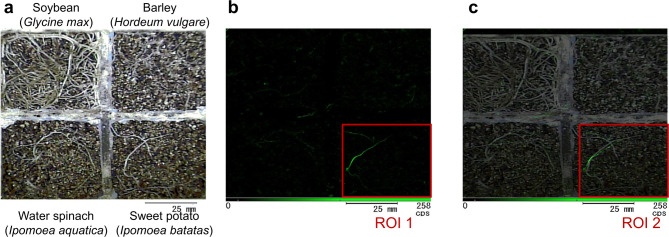
X-ray fluorescence mapping images of roots among different species with vermiculite background in X-ray fluorescence mapping test. (**a**) Optical image of the roots. (**b**) Mapping image of potassium. (**c**) An overlapping of the optical image and the K map to show the element’s location on the root architecture. Regions of Interest (ROI) were selected to quantify K intensity on the root (ROI 1) and the vermiculite background (ROI 2).

In the vermiculite medium, sweet potato roots exhibited stronger K signals compared to the medium (Fig. 3b), with peak values exceeding 150 cps on the calibrated intensity scale, showing a clear distribution pattern that was consistent with the root architecture (Fig. 3c). For water spinach, only part of the roots showed stronger K signals than the medium (Fig. 3c). In contrast, barley and soybean exhibited negligible differences in K signals (Fig. 3b), making the K distribution along the roots less distinct (Fig. 3c). Such an interspecific difference was not observed in the other elements (Na, S, and Zn) (Fig. S2a–c).

#### Mixed-culture experiment

**Fig. 4:**
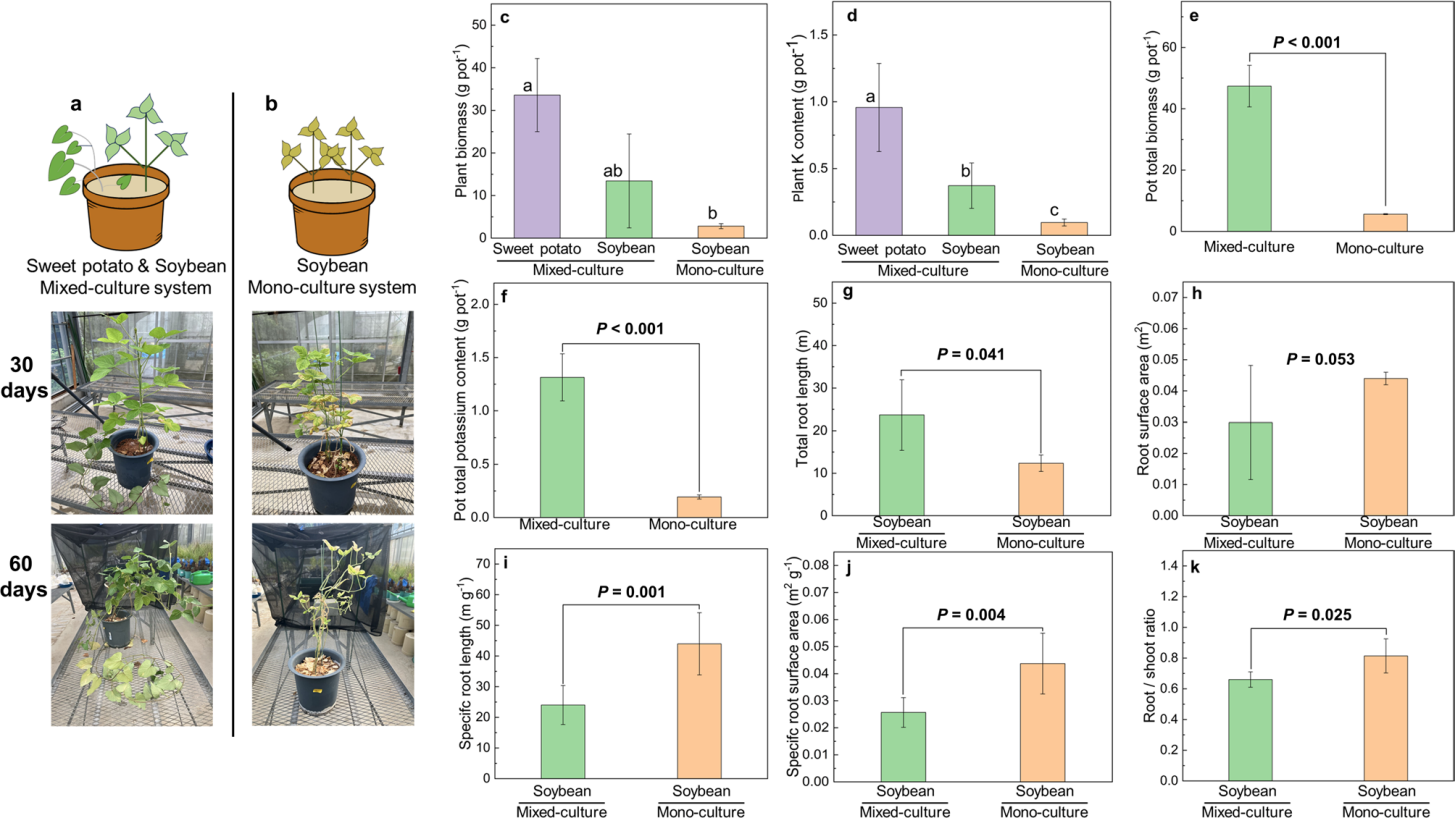
Diagrams and photographs of sweet potato & soybean mixed-culture system (**a**) and soybean mono-culture system (**b**) after 30 and 60 days of growth in vermiculite-only medium in mixed-culture experiment in 2024. Plant biomass (**c**) and K content (**d**), pot total biomass (**e**) and K content (**f**), and root morphology (total root length (**g**), root surface area (**h**), specific root length (**i**), specific root surface area (**j**), root/ shoot ratio (**k**) in sweet potato & soybean (S&P mixed) and soybean mono-culture (S&S mono) system in mixed-culture experiment in 2024 with vermiculite-only medium. The growth period was 60 days. In panels (**c**) and (**d**), different letters indicate significant differences (*P* < 0.05) in individual plant biomass and K content among the different plant species, respectively. In panels (**e**) to (**k**), independent *t*-test was used to determine the significant differences between sweet potato & soybean (S&P mixed) and soybean mono-culture (S&S mono) system under the same growth environment.

Soybean exhibited distinct visual appearance in the sweet potato and soybean mixed-culture system compared to the soybean monoculture system (Fig. 4). At 30 days after transplanting, soybeans in the mixed-culture system displayed normal growth (Fig. 4a), whereas those in the mono-culture system experienced increased leaf drop and yellowing at the leaf margins (Fig. 4b). At 60 days after transplanting, soybeans in the mixed-culture system continued to grow normally (Fig. 4a). In contrast, those in the mono-culture system exhibited pronounced leaf wilting and discoloration, stem bending, and reduced biomass (Fig. 4b).

In the sweet potato-soybean mixed-culture system, each soybean plant accumulated 0.40 g of K from the vermiculite-only medium. In comparison, the soybean mono-culture system resulted in an accumulation of approximately 0.10 g of K per plant (Fig. 4c, d). Furthermore, the mixed-culture system demonstrated significantly higher accumulation of K (1.31 g), and biomass (46.3 g) compared to the soybean mono-culture system, which accumulated 0.19 g K pot^−1^, and 5.62 g biomass pot^-1^ (Fig. 4e, f).

Soybean exhibited a greater total root length when cultivated in a mixed-culture system with the sweet potato (Fig. 4g). While no significant differences were observed between the two culturing systems regarding root surface area (Fig. 4h). Additionally, the specific root length (SRL) and specific root surface area (SRA) of the soybean were lower in the sweet potato soybean mixed-culture system compared to the soybean mono-culture system (Fig. 4i, j). Besides, root/shoot ratio of soybean in the mixed-culture system was significantly lower than that in mono-culture system (Fig. 4k).

**Fig. 5:**
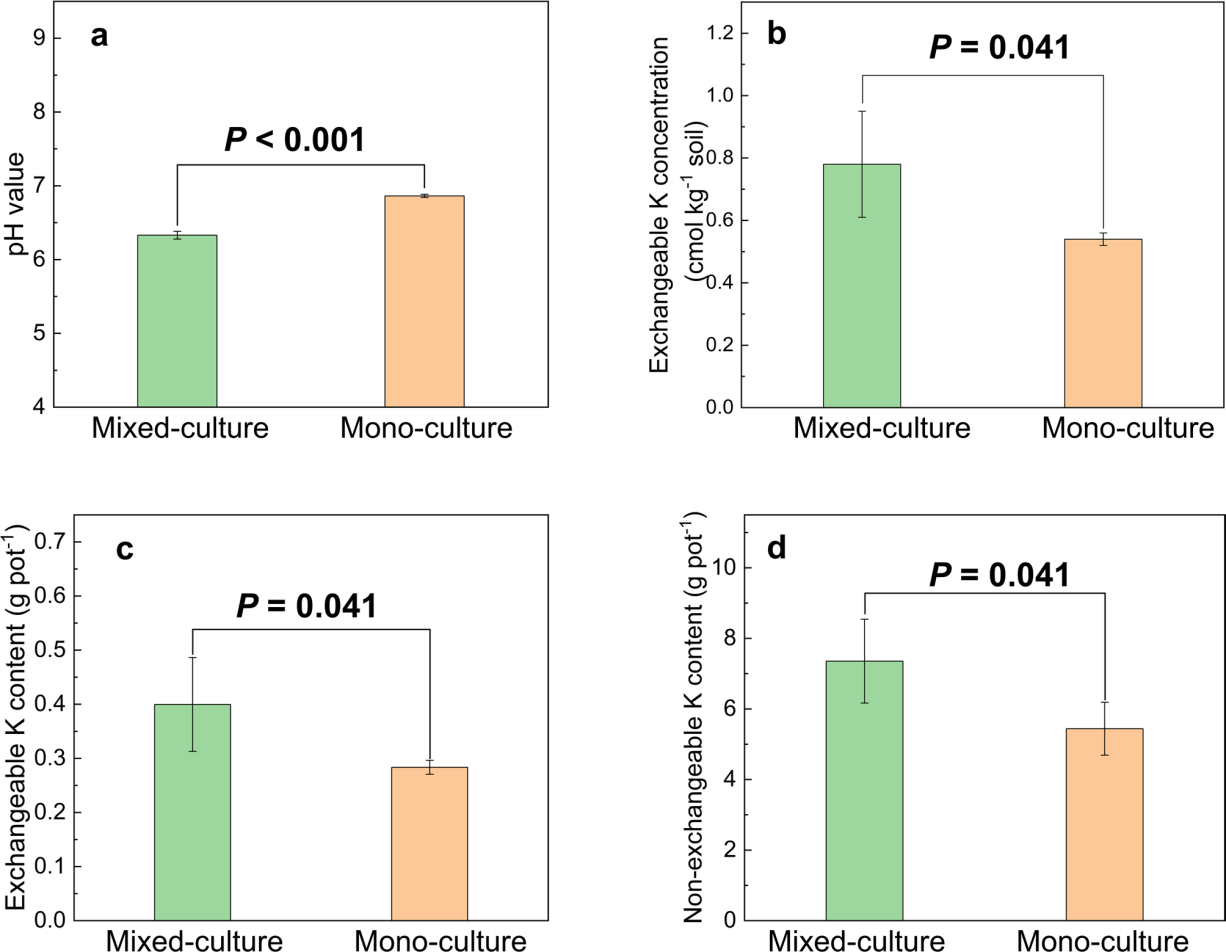
pH value (**a**), exchangeable K concentration (**b**), exchangeable K content (**c**), non-exchangeable K content (**d**) of growth medium in sweet potato & soybean (S&P mixed) and soybean & soybean (S&S mono) system in mixed-culture experiment in 2024 with vermiculite-only medium. The growth period was 60 days. Independent *t*-test was used to determine the significant differences between sweet potato & soybean (S&P mixed) and soybean mono-culture (S&S mono) system under the same growth environment.

From the perspective of vermiculite nutrients after growth, Fig. 5 shows that exchangeable K concentration in vermiculite-only medium reached 0.78 cmol kg^−1^. Besides, the exchangeable (0.4 g pot^−1^) and non-exchangeable (7.35 g pot^−1^) K content in the growth medium of the sweet potato-soybean mixed-culture system was significantly higher than that in the soybean mono-culture system. Additionally, the pH value of the growth medium in the mixed-culture system (pH = 6.33) was lower than that in the soybean mono-culture system (pH = 6.86) and the original vermiculite medium (pH = 7).

#### Buffer solution pH incubation experiment

As shown in Supplementary Fig. S4, exchangeable K concentrations in vermiculite varied with pH treatment. Incubation at pH 6.0 and pH 6.5 resulted in exchangeable K levels of 597.11± 42.10 mg kg^−1^ and 530.00 ± 87.03 mg kg^−1^, respectively, representing increases of 39% and 24% compared to the concentration measured at pH 7.0.

## Discussion

Previous research has shown that some *Ipomoea* species can alter soil nutrient availability, such as water spinach enhancing silicon access for rice^14,28^. Our study revealed that sweet potato, a close relative, demonstrated a powerful capacity to uniquely promote the release of K from mineral sources like vermiculite which is not accessed by other crop species (Fig. 2). Furthermore, sweet potato enhances K availability in vermiculite medium and K and biomass accumulation of neighboring soybean during 60 days of mixed culturing (Figs. 4 and 5). This trait suggests a possible avenue for developing sustainable intercropping systems that reduce the reliance on chemical K fertilizers by better utilizing native soil K reserves and alternative K-bearing minerals with similar 2:1 structure and fixed interlayer K ions.

### Ipomoea plants, in particular sweet potato, can utilize vermiculite as available K source

Consistent with the previous report^19^, results in growth response experiment in 2023 also showed that sweet potato exhibited a negligible response in terms of biomass and K accumulation to fertilizer K input in the mixed medium of Andosol and vermiculite (Fig. 1a). However, this lack of response was observed not only in sweet potato but also in soybean and barley (Fig. 1c, d), indicating that the mixed medium provided sufficient available K for these plants even without K fertilization. In contrast, water spinach displayed a significant response, with 1.4-fold higher biomass and 2.1-fold higher K accumulation at 1 g K pot^−1^ compared to no K input (Fig. 1b, f). As a semi-aquatic plant, water spinach is known to accumulate excessive potassium under well-watered conditions^29^, leading to “luxury consumption” where surplus K is stored in cellular vacuoles (Gobert et al. 2007). These results indicated that, except for water spinach, the plants examined likely met their K demands solely from the medium, without the need for additional K-fertilizer.

When the plants were grown in a vermiculite-only medium without K fertilizer input, both soybean and barley showed poor biomass due to negligible K accumulation (Fig. 1c, d), indicating that the available K was extremely limited for these plants in this condition. Therefore, we can answer question (1) by asserting that the available K source was likely derived from Andosol, which fully satisfied the K demand in the mixed medium without K fertilization. In contrast, sweet potato and water spinach were able to accumulate K to remarkable extents in the vermiculite-only medium without K fertilizer input (Fig. 1a, b), revealing that both *Ipomoea* species could utilize not only Andosol but also vermiculite as an available K source.

Moreover, we confirmed that sweet potato can accumulate K in the roots developed in vermiculite as a sole K source, as evidenced by X-ray fluorescence mapping, which showed intensive K signal aligning with the root architecture (Fig. 3). This observation was further supported by the finding that sweet potato roots in the vermiculite-only medium exhibited the highest K concentration in growth response experiment (Table S2). This finding is noteworthy because K-bearing minerals, such as vermiculite, are frequently regarded as ineffective K sources for plants due to their slow release rates, which typically result in minimal yield response compared to soluble K fertilizers^30,31,6^. Further quantitively X-ray fluorescence mapping could be applied in future research to test the different K concentration of growth medium before and after *Ipomoea* plants cultivation.

### Unique K acquisition capacity of sweet potato is likely due to the availability of interlayer K in vermiculite

The result is consistent with the unique K acquisition capacity being associated with the ability of *Ipomoea* plants to promote the release of interlayer K reserves in vermiculite. In the absence of K fertilizer inputs, sweet potato accumulated 1.05 g K pot^−1^ during 60 days of growth in the vermiculite-only medium (Table 3). Despite that, exchangeable K in the medium decreased by 0.06 cmol kg^−1^, 0.15 g in pot level, suggesting that as plants consumed the readily available exchangeable K, this pool was continuously replenished by a large release of K from the slowly available K pool (Fig. 2). A mass balance calculation indicates that this buffering process accounted for over 85% of the total K accumulated by sweet potato and water spinach, highlighting the critical role of these slowly available K pool in the vermiculite-only medium (Fig. 2). This phenomenon was also observed in the mixed soil medium and may be attributed to the acidified rhizosphere of sweet potato under low K inputs (Table 2). Similar phenomena have been reported in ruzigrass (*Urochloa ruziziensis*)^32^ and tobacco (*Nicotiana tabacum*)^33^. In contrast, soybean and barley showed poor K accumulation and were unable to acquire sufficient K from slowly available K pool in the vermiculite-only medium (Fig. 2).

Vermiculite’s 2:1 layered structure, characterized by fundamental layers where two tetrahedral (primarily silica) sheets sandwich a central octahedral (primarily alumina/magnesia) sheet, creates interlayer spaces that facilitate the fixation of K ions^34^. Owing to this unique structure, vermiculite is considered as a buffer for K availability by adsorbing excessive K from fertilizer inputs and releasing it under K-deficient conditions^34,35,36^. In previous research, ground micas, the primary minerals of vermiculite, have been applied to highly weathered soils, resulting in increased soil K availability^37^. However, the potential of vermiculite as K source appears to be interspecific. In response to question (2), *Ipomoea* plants, which had strong access to slowly available K pool, were able to utilize vermiculite as K source in the absence of chemical K fertilization in the mixed soil medium (Fig. 2). Conversely, for plants with weak K uptake capacity, such as legumes^38,39^, vermiculite provides only a minimal K supply.

### Sweet potato improved soybean K nutrition through the rhizosphere effect

The beneficial rhizosphere effect of sweet potato was confirmed in our mixed-culture experiment, where co-cultured soybean thrived while mono-cultured soybean showed symptoms of severe K deficiency (Figs. 4 and 5). In the mixed-culture experiment in 2024, soybean grown in a mono-culture system exhibited poor K uptake and abnormal growth under different levels of K inputs in the vermiculite-only medium after 60 days of growth (Fig. 4b). However, when co-cultured with sweet potato, K accumulation of soybean significantly increased (0.40 g plant^−1^) compared to mono-culture (0.09 g plant^−1^) (Fig. 4b). At the pot level, sweet potato and soybean mixed-culture system showed greater K accumulation and biomass than soybean grown in mono-culture (Fig. 4e, f). These findings suggest that interspecific interactions within the rhizosphere play a crucial role in enhancing nutrient availability in mixed-culture systems, a perspective supported by previous research^40,41^.

In this study, enhanced K uptake in the mixed-culture system was likely driven by rhizosphere-mediated K release from vermiculite initiated by sweet potato (Fig. 2), rather than solely by the high K uptake affinity of its roots, as reported by Jiang et al.^12^ and Wang et al.^13^. In our study, an increase in both exchangeable and non-exchangeable K content was observed in the mixed-culture system (Fig. 5), suggesting that the vermiculite may release more interlayer potassium by the rhizosphere effect of sweet potato in vermiculite. The release of non-exchangeable K is believed to be closely linked to secretion of root exudates, especially organic acids^33,42^. During this process, root exudates accelerate the releasing of interlayer potassium ions and promote the transformation of fixed K into exchangeable forms^43,44,33^. While the role of root exudates in sweet potato P acquisition has been considered minor (Minemba, D. 2020), and their effect on K mobilization from minerals has remained unclear.

Along with the secretion of organic acids, protons are also released to the rhizosphere to maintain the membrane potential balance of root cells^45^. However, the influence of rhizosphere pH on soil K availability remains unclear^46,47^. In the sweet potato and soybean mixed-culture system in our experiment, a decrease in pH value (pH = 6.33) was observed compared to the soybean mono-culture system (pH = 6.86), accompanied by a 41% increase in exchangeable K concentration (Fig. 5a). When vermiculite was incubated for 24 hours in buffer solution with a pH range from 6.0 to 7.0, similar variations in exchangeable K concentration are also observed (Supplementary Fig. S4). Interestingly, the changes in pH value observed in the vermiculite incubation experiment accounted for only 65% of the variation in vermiculite exchangeable K concentration. This indicates that covariate covariance, such as the effect of organic acids, should be considered within the context of the mixed-culture system. Overall, these findings suggest that sweet potato not only sustained a high K acquisition capacity in vermiculite of itself but also enhanced K release of vermiculite and K availability throughout the entire rhizosphere. Our results provide evidence that sweet potato is associated with a decrease in the pH of the vermiculite medium and with increased K release from vermiculite. However, this evidence remains indirect, and the proposed rhizosphere acidification mechanism is hypothesized rather than directly demonstrated. Direct measurement of rhizosphere organic acid exudation and proton flux were not conducted in the present study.

Biomass allocation and root morphology likely supported the observation that K nutrition in soybean was improved by mixed-culture with sweet potato (Fig. 4). Soybean exhibited a higher root/shoot ratio in mono-culture compared to the mixed-culture system (Fig. 4k). This phenomenon is believed to be associated with plant K deficiency as plants often allocate more resources to root growth to acquire K nutrients^48^. Compared to mixed-culture soybean, mono-culture ones developed smaller and finer roots (higher SRL and SRA) (Fig. 4i, j). Such root characteristics are commonly observed in nutrient-poor or dry environments, where plants maximize nutrient and water uptake through root system development^49^. In addition, soybean in the mixed-culture system exhibited increased total root length compared to mono-culture soybean (Fig. 4g), a trait associated with enhanced drought tolerance and yield potential^50,51^.

While the total K accumulation of soybean in the K-deficient mono-culture system was drastically lower than that in the mixed-culture system, the K concentration in its tissues was similar. This measured tissue K concentration (3.4% in stem tissue, 2.5% in root tissue) (Fig. S3) falls within the sufficiency range reported for soybean (2.0–3.5% in stem tissue, around 2% in root tissue) at this growth stage^52^. Similar phenomenon has also been observed in barley plants grown in vermiculite-only medium (Table S2), with aboveground tissue K concentration (3.5%) have been considered normal (2.8–4.7%) compared to previous research^53^. This paradox can be explained by the hypothesis that there is a severe inhibition of biomass production under K deficient environment. It has been demonstrated that growth is a more sensitive response to K deficiency than the reduction of tissue K concentration^54^. This finding is consistent with foundational principles where nutrient deficiencies limit yield long before tissue concentrations drop below a critical level^55^, indicating that total K accumulation was a more accurate indicator of plant K stress in this research.

## Conclusion

This study provides evidence that sweet potato and water spinach, both belonging to genus *Ipomoea*, can promote the release of K from slowly available K pool in vermiculite, which was not accessed by soybean and barley. As a result, both *Ipomoea* species exhibited normal growth in vermiculite even without K fertilizer, while soybean and barley showed poor growth due to severe K-deficiency. X-ray fluorescence mapping further revealed substantial K accumulation in the roots of sweet potato grown in vermiculite, even after just 7 days.

These findings prompted an investigation into whether the severe K deficiency observed in soybeans could be alleviated by mixed-culturing with sweet potato. The results demonstrated that mixed-culture with sweet potato significantly enhanced K accumulation and biomass in soybean compared to mono-culture. These improvements are likely attributable to a rhizosphere effect of sweet potato, which results in a decrease in the pH value of the vermiculite medium, facilitating the release of interlayer K from vermiculite. While these results from two-season pot experiments are promising, further validation through multi-season, long-term field experiments are crucial to confirm these findings under complex agricultural conditions and to evaluate the practical and economic benefits of this intercropping strategy. Future studies incorporating direct measurement of rhizosphere proton flux, root-exudate composition, and mineralogical changes (e.g. by X-ray diffraction (XRD) or scanning electron microscopy (SEM)) should be conducted to elucidate the mechanisms underlying the rhizosphere effect of sweet potato and other *Ipomoea* species, which may contribute to the utilization of vermiculite as a novel K resource, thereby reducing the reliance on chemical K fertilizers.

## Supplementary Information

Below is the link to the electronic supplementary material.

Supplementary Material 1

## Data Availability

The authors confirm that the data supporting the findings of this study are available within the article and its supplementary material.
