# Supplementary material for: K acquisition from vermiculite by sweet potato also improves K nutrition in neighboring plants
Source: Sci Rep. 2026 Jun 10;16:20773. doi: 10.1038/s41598-026-56682-4 (PMC13338254; doi:10.1038/s41598-026-56682-4)
Supplement: Supplementary file 1 — Supplementary Material 1 [file 41598_2026_56682_MOESM1_ESM.docx]

**K acquisition from vermiculite by sweet potato also improves K nutrition in neighboring plants**

Fan Mo^1^*, Yan Yi^1,2,3^, Katsuya Yano^1^

^1^ Laboratory of Crop Science, Graduate School of Bioagricultural Sciences, Nagoya University, Nagoya 464-8601, Aichi, Japan

^2^ Panxi Crops Research and Utilization Key Laboratory of Sichuan Province, School of Agricultural Science, Xichang University, Xichang 615013, Sichuan, China

^3^ International Research Fellow of Japan Society for the Promotion of Science

The following Supporting Information is available for this article:

Table S1 Physicochemical for the vermiculite used in the experiment.

| **Vermiculite physicochemical properties** | |
| --- | --- |
| SiO_2_ (%) | 51.0 |
| Fe_2_O_3_ (%) | 13.0 |
| AL_2_O_3_ (%) | 13.0 |
| TiO_2_ (%) | 1.3 |
| CaO (%) | 3.0 |
| MgO (%) | 11.0 |
| K_2_O (%) | 3.1 |
| pH | 7.0 |
| Particle size (mm) | 1.0-3.0 |

**Table S2** Tissue K concentration in sweet potato, barley, water spinach, and soybean in growth response experiment in 2023 with vermiculite-only medium. Different letters indicate significant difference among different plant species in relation to stem and root K concentration (*P* < 0.05).

| Species | Leaf K concentration (%) | Stem K concentration^†^ (%) | Root K concentration (%) | Tuber K concentration (%) |
| --- | --- | --- | --- | --- |
| Sweet potato | 5.02±0.85 | 2.53±0.57^a^ | 5.70±0.87^a^ | 1.46±0.28 |
| Water spinach | 6.71±1.45 | 5.03±1.11^b^ | 3.88±1.02^b^ | n.a. |
| Barley | n.a. | 3.40±1.11^ab^ | 1.90±0.29^c^ | n.a. |
| Soybean | n.a. | 3.71±0.47^ab^ | 2.53±0.09^bc^ | n.a. |

Note: ^†^  Biomass yield was reduced in the vermiculite-only treatment owing to K deficiency-induced wilting in both soybean and barley. Consequently, leaf and stem tissues were combined and are hereafter referred to as “stem”.

Table S3 Coefficient of variation (CV, %) for the values reported in Tables 2

| Species | Treatment (g K pot-1) | pH value | Exchangeable K concentration (cmol kg-1) | Exchangeable K content (g pot-1) | Non-exchangeable K content (g pot-1) | Changes in slowly available K pool (g pot-1) |
| --- | --- | --- | --- | --- | --- | --- |
| Sweet potato | 0 | 1.1 | 10.3 | 9.4 | 23.3 | 20.9 |
| Sweet potato | 0.25 | 0.7 | 17 | 17.2 | 15.1 | 15.5 |
| Sweet potato | 0.5 | 1.2 | 14 | 14.3 | 33 | 14.7 |
| Sweet potato | 1 | 1.1 | 13.7 | 14.5 | 38.1 | 29.2 |
| Water spinach | 0 | 1.6 | 15.7 | 15.9 | 17.8 | 25.4 |
| Water spinach | 0.25 | 1.4 | 3.8 | 4.2 | 36.2 | 21 |
| Water spinach | 0.5 | 0.5 | 9.4 | 9.7 | 36.5 | 18.7 |
| Water spinach | 1 | 1.1 | 6.3 | 6.9 | 9.9 | 3.8 |
| Barley | 0 | 0.5 | 9.1 | 8.9 | 7.8 | 9.9 |
| Barley | 0.25 | 1.8 | 2.7 | 3 | 8.3 | 62.5 |
| Barley | 0.5 | 1.4 | 12.3 | 12.4 | 11.1 | 100^†^ |
| Barley | 1 | 1.6 | 32.7 | 32.4 | 9.2 | 83.6 |
| Soybean | 0 | 2.2 | 31.1 | 31.1 | 28.7 | 14 |
| Soybean | 0.25 | 2 | 23.1 | 22.5 | 20.6 | 9.4 |
| Soybean | 0.5 | 9.4 | 12.7 | 12 | 21.6 | 7.7 |
| Soybean | 1 | 2.5 | 28.3 | 29.2 | 13.6 | 39 |

^†^CV (%) = (SD / mean) × 100, calculated from the mean and SD values reported in Table 2 (n = 4 per cell). The high CV values for changes in slowly available K in barley are mathematically inflated because the absolute mean values for this metric are small in this species; the underlying standard deviations are comparable to those of other species.


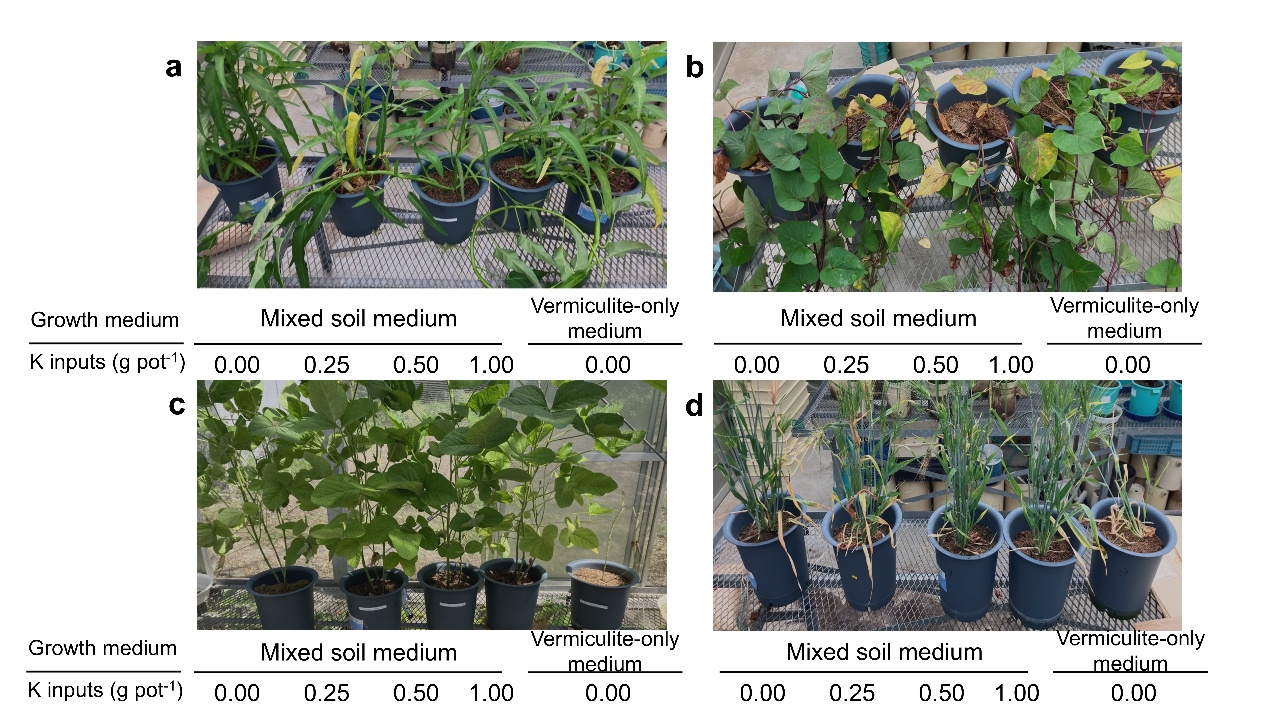


**Fig. S1** Photograph of water spinach **(a)**, sweet potato **(b)**, soybean **(c)**, and barley **(d)** in growth response experiment in 2023. All the plants were grown in different growth medium with different K inputs after 60 days of growth.


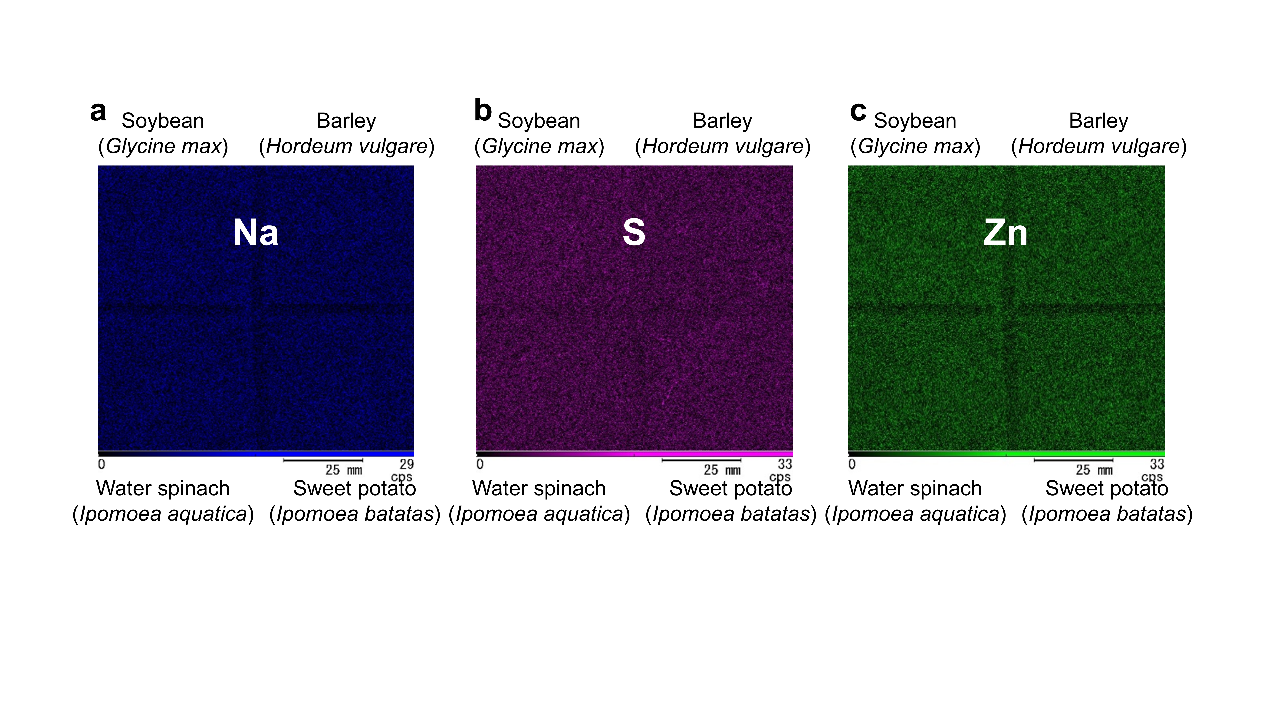


**Fig. S2** X-ray fluorescence mapping images of roots among different species with vermiculite background in X-ray fluorescence mapping test. **(a)** Mapping image of sodium. **(b)** Mapping image of sulfur. **(c)** Mapping image of zinc.

**
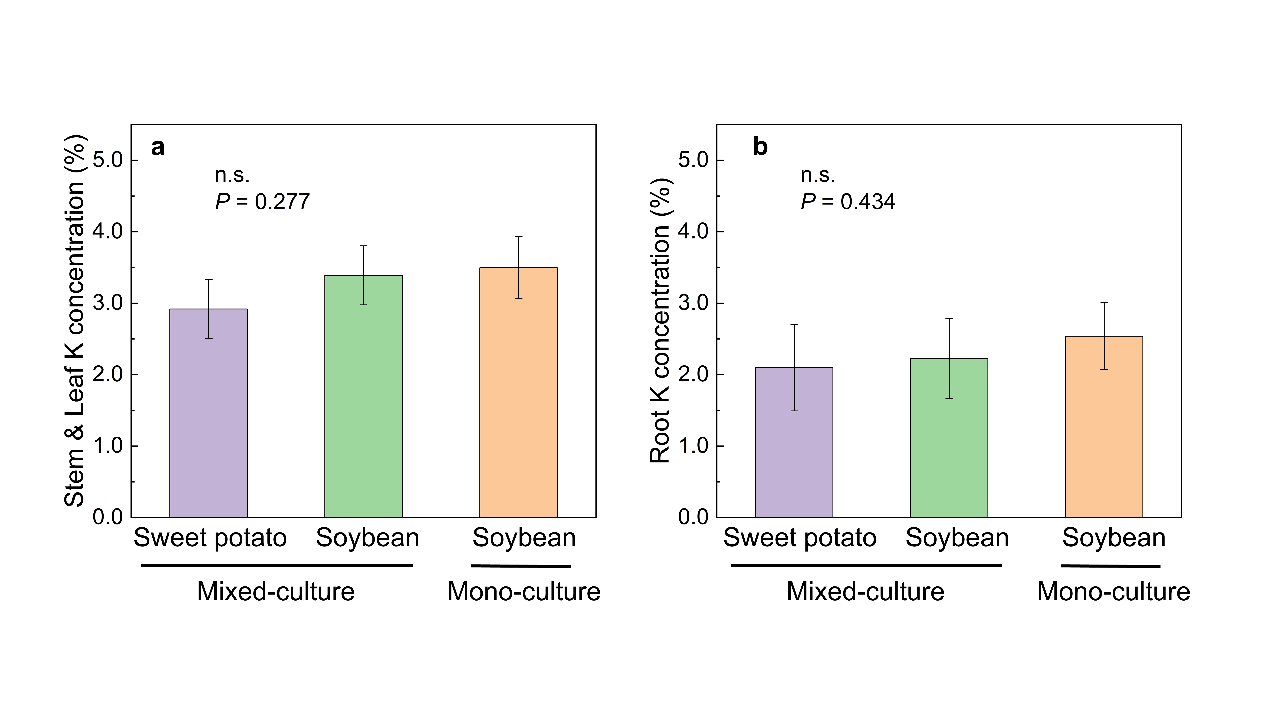
**

**Fig. S3** Stem and leaf **(a)** and root **(b)** K concentration in sweet potato and soybean in different growth system in mixed-culture experiment in 2024. Biomass yield was reduced in the vermiculite-only treatment owing to K deficiency-induced wilting in both soybean and barley. Consequently, leaf and stem tissues were combined and are hereafter referred to as 'Stem & Leaf'. Different letters denote statistically significant differences among the plant species according to a Tukey's HSD post-hoc test (*P* < 0.05).


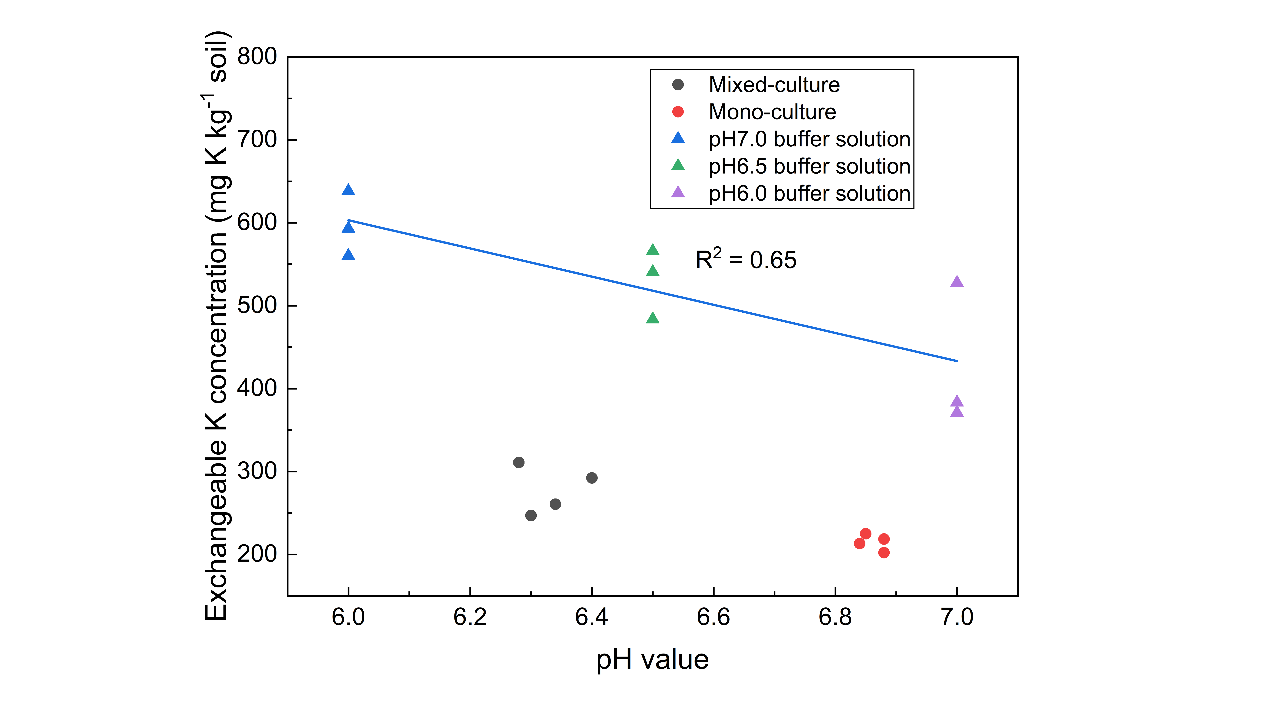


**Fig. S4** Soil exchangeable K content of vermiculite after 60 days of growth in greenhouse with different culture system and 24 hours of incubation by 1M buffer solution composed of (Na_2_HPO_4_/NaH_2_PO_4_) at varying pH levels.
